# First clinical use of the kisspeptin analog TAK-683 in the treatment of a follicular cyst in a goat: case report

**DOI:** 10.1007/s11259-026-11141-3

**Published:** 2026-03-12

**Authors:** Gökhan Uyanık, Murat Yüksel, Filiz Kara, Ahmet Gözer, Murat Abay

**Affiliations:** 1 Faculty of Veterinary Medicine, Department of Obstetrics and Gynaecology, Hatay Mustafa Kemal University, Antakya, Hatay 31001 Türkiye; 2 Institute of Health Sciences, Department of Veterinary Obstetrics, Gynaecology and Artificial Insemination, Hatay Mustafa Kemal University, Antakya, Hatay 31001 Türkiye; 3 Faculty of Veterinary Medicine, Department of Obstetrics and Gynaecology, Erciyes University, Kayseri, Talas 38280 Türkiye

**Keywords:** Ovarian cyst, Reproductive endocrinology, Small ruminant, Ultrasonography

## Abstract

This case report presents the therapeutic response to TAK‑683, a long-acting kisspeptin analog, in the treatment of a follicular cyst that developed following a conventional estrus synchronization protocol in a goat during the non-breeding season. A two-year-old lactating Aleppo goat showing persistent nymphomaniac behaviour after estrus synchronization was diagnosed with an ovarian follicular cyst by transrectal ultrasonography. The left ovary contained a 17.7 mm thin-walled anechoic cyst, while serum estradiol (14.5 pg/mL) was elevated and progesterone (1.6 ng/mL) was low. The goat was treated with a single subcutaneous dose of the kisspeptin analog TAK-683 (5 µg, Day 0). Therapeutic efficacy was assessed by monitoring ovarian ultrasonography and serum hormones on Days 3, 10, 13, and 17. Following treatment, the cyst underwent luteinization accompanied by a rise in progesterone (peak 6.4 ng/mL) and a decline in estradiol to basal levels, alongside normalization of behaviour. Color Doppler assessment confirmed progressive luteal development and vascularization, followed by natural luteolysis on Day 17 and resumption of follicular activity. In conclusion, this is the first clinical report demonstrating the successful use of TAK‑683 in resolving a follicular cyst in goats. These findings suggest that TAK‑683, beyond its ovulation-inducing role, may offer a promising therapeutic alternative to GnRH agonists for managing reproductive pathologies in veterinary gynaecology.

## Highlights

First report of follicular cyst treatment in a goat with TAK-683.

5 µg of TAK-683 induced luteinization and normalized hormone levels.

TAK-683 may offer an alternative to GnRH agonists in follicular cyst treatment.

## Introduction

Follicular cysts are one of the major reproductive disorders contributing to infertility in goats, reportedly accounting for approximately 15–20% of all reproductive problems (Medan et al. 2004; Khan et al. 2016; Samuel et al. 2025). In goats, a follicular cyst is defined as a fluid-filled, thin-walled follicle larger than 10 mm in diameter that persists for more than 10 days in the absence of a functional corpus luteum (CL) and is typically associated with prolonged or nymphomaniac estrus behaviour (Medan et al. 2004; Khan et al. 2016). For diagnosis, the presence of clinical symptoms and elevated serum estradiol (E_2_) concentrations, in combination with ultrasonographic findings, are considered the most reliable criteria (Medan et al. 2004; Simões et al. 2009). The most widely adopted therapeutic approach for follicular cysts in goats involves the administration of gonadotropin-releasing hormone (GnRH) analogs to induce luteinization or ovulation of the cystic follicle (Medan et al. 2004; Khan et al. 2016). GnRH administration stimulates luteinizing hormone (LH) secretion from the pituitary, facilitating the transformation of the anovulatory cystic follicle into a functional structure. In the literature, GnRH treatment in goats has been associated with behavioural normalization, reduction in cyst diameter, and an increase in serum progesterone (P_4_) concentrations (Khan et al. 2016).

However, therapeutic responses are not always consistent, and cases of follicular cysts unresponsive to GnRH treatment have been reported (Kumar et al. 2022). This variability suggests that cyst persistence may sometimes originate from upstream neuroendocrine dysregulation rather than isolated pituitary insufficiency (Sarath et al. 2025). In this context, kisspeptin has emerged as a key upstream regulator of reproductive neuroendocrine function (Beltramo and Decourt 2018). Acting as a principal hypothalamic mediator of GnRH release, kisspeptin plays a crucial role in the control of gonadotropin secretion and ovulation. Within the hypothalamus, kisspeptin neurons interact with neurokinin B and dynorphin to regulate pulsatile GnRH secretion (Masumi et al. 2022). Stress-related factors, negative energy balance, seasonal influences, and lactation-associated physiological load may suppress kisspeptin activity, leading to altered GnRH/LH secretion and subsequent anovulation, mechanisms recognized as contributors to follicular cyst persistence (Yeo and Colledge 2018). Under these conditions, GnRH analogs can bypass this upstream dysfunction, which may explain inadequate treatment responses in some cystic cases (López-Gatius and López-Béjar 2002).

In recent years, kisspeptins have been reported as strong alternatives to GnRH agonists (Beltramo and Decourt 2018; Uyanık et al. 2024). Kisspeptins directly stimulate hypothalamic GnRH neurons, inducing physiological gonadotropin release in a manner aligned with biological rhythms. Compared to GnRH agonists, kisspeptins are considered a safer option due to their low risk of receptor desensitization and other side effects (Uyanık et al. 2024). Furthermore, the presence of kisspeptin receptors in ovarian follicles and oocytes suggests additional local autocrine/paracrine actions that may support follicular development and oocyte maturation, conferring a broader spectrum of activity to kisspeptin analogs (Masumi et al. 2022). This favourable physiological profile has driven clinical interest in long acting kisspeptin analogs capable of inducing a predictable LH surge and timed ovulation with a single dose. One such analog, TAK‑683, has been extensively studied in goats, where single-dose administration was shown to significantly increase circulating follicle-stimulating hormone (FSH) and LH concentrations by stimulating endogenous GnRH secretion (Goto et al. 2014; Kanai et al. 2017). Notably, Kanai et al. (2017) reported that a 5-µg dose of TAK‑683 in synchronized cyclic goats triggered an LH surge lasting approximately 10 h, followed by ovulation on day 3 and accelerated CL development.

These findings suggest that TAK‑683 may serve not only as an ovulation inducer but also as a potential therapeutic agent for ovarian pathologies such as follicular cysts representing an alternative to GnRH agonists. However, to the best of our knowledge, no previous clinical application of kisspeptin analogs for the treatment of follicular cysts in goats has been documented. This case report aims to present, for the first time, the diagnosis and successful treatment of a follicular cyst using TAK‑683 in a lactating Aleppo goat during the non-breeding season, detailing the diagnostic process, therapeutic protocol, endocrine responses, and ovarian dynamics. The findings offer preliminary insight into the potential role of kisspeptin analogs as an effective alternative to GnRH agonists in veterinary gynaecology.

## Case presentation

### History

**Fig. 1:**
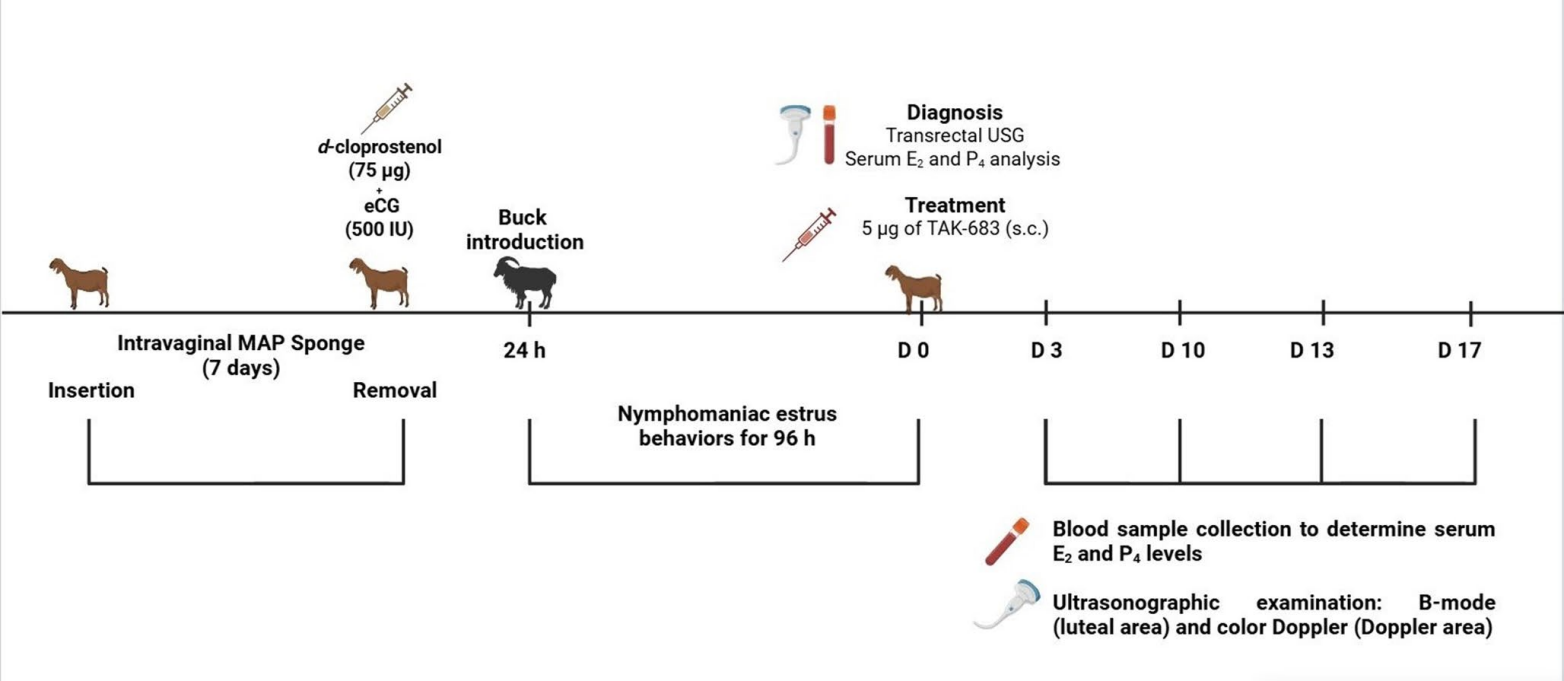
Schematic representation of estrus synchronization and subsequent cyst treatment in an Aleppo goat outside the breeding season. An intravaginal MAP sponge was applied for 7 days and removed with *d*-cloprostenol (75 µg) and eCG (500 IU), followed 24 h later by buck introduction. Nymphomaniac estrus behaviours persisted for 96 h, after which a cystic follicle was diagnosed on D0 by ultrasonography and serum E_2_/P_4_ analysis. A single 5 µg TAK-683 injection was administered, and follow-up on D3, D10, D13, and D17 included hormone assays and ultrasonographic (B-mode and Doppler) evaluation

During the non-breeding season (March 2025), a conventional synchronized estrus induction protocol was implemented in 45 lactating Aleppo goats (aged 3–5 years, mean body weight 44.46 ± 0.44 kg and mean body condition score 2.62 ± 0.06) from a commercial dairy goat farm located in the Reyhanlı district of Hatay, Türkiye (36°15’12.2"N, 36°32’02.6"E; altitude: 138 m). Based on routine transrectal ultrasonographic examinations performed prior to the initiation of the protocol, it was confirmed that the goats were clinically and reproductively healthy before being included in the synchronization protocol. The protocol involved the insertion of a commercial intravaginal sponge containing medroxyprogesterone acetate (MAP) (Esponjavet^®^, Hipra, Spain) for 7 days, followed by an intramuscular injection of 500 IU eCG (Oviser^®^, Hipra, Spain) and 75 µg *d*-cloprostenol (Senkrodin^®^, Vetaş, Türkiye) at sponge removal. Twenty-four hours after sponge withdrawal, fertile bucks were introduced into the herd for natural mating at a buck-to-doe ratio of 1:9. Estrus activity gradually declined across the herd and had completely ceased by 72 h post-sponge removal. However, one goat exhibited persistent estrus behaviour beyond the expected period, characterized by pronounced nymphomaniac activity, decreased lactational output, and reduced feed intake. Due to the continuation of these clinical signs for 96 h, the goat was separated from the flock and underwent a detailed clinical and gynaecological evaluation (Fig. 1).

### Clinical examination and diagnosis

**Fig. 2:**
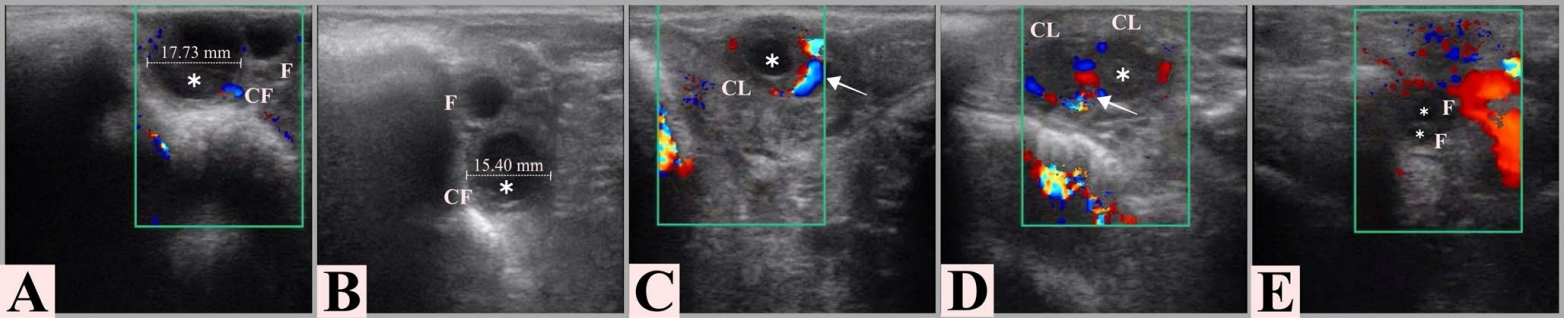
Representative B-mode and color Doppler ultrasound images of the suspected cystic follicle on day 0 (**A**) and during subsequent monitoring following a single subcutaneous administration of 5 µg of TAK-683. Transrectal ultrasonographic evaluations were performed on days 3 (**B**), 10 (**C**), 13 (**D**), and 17 (**E**) post-treatment to monitor follicular and luteal dynamics. Legend: (*) anechoic cavity; (→) Doppler signal; CL: corpus luteum; F: follicle; CF: cystic follicle

Clinical evaluation indicated that the goat (2 years old, multiparous, 45 kg, body condition score: 2.5) had stable vital parameters and showed no signs of systemic disease. Examination of the reproductive tract revealed vulvar edema and hyperaemia of the vaginal mucosa. Transrectal ultrasonography (SonoSite M-Turbo, Fujifilm, USA, 5–10 MHz linear rectal probe) showed mild uterine edema. In the left ovary, a thin walled (2.10 mm), anechoic follicular cyst measuring 17.73 mm in diameter was identified together with an additional 7.79 mm follicle (Fig. 2A), while three small follicles (< 5 mm) were observed in the right ovary, with no CL detected in either ovary. To determine serum hormone concentrations (E_2_ and P_4_), approximately 8 mL of blood was collected via jugular venipuncture into plain vacuum tubes containing clot activator gel (Vacusera^®^, Disera, Türkiye).

**Fig. 3:**
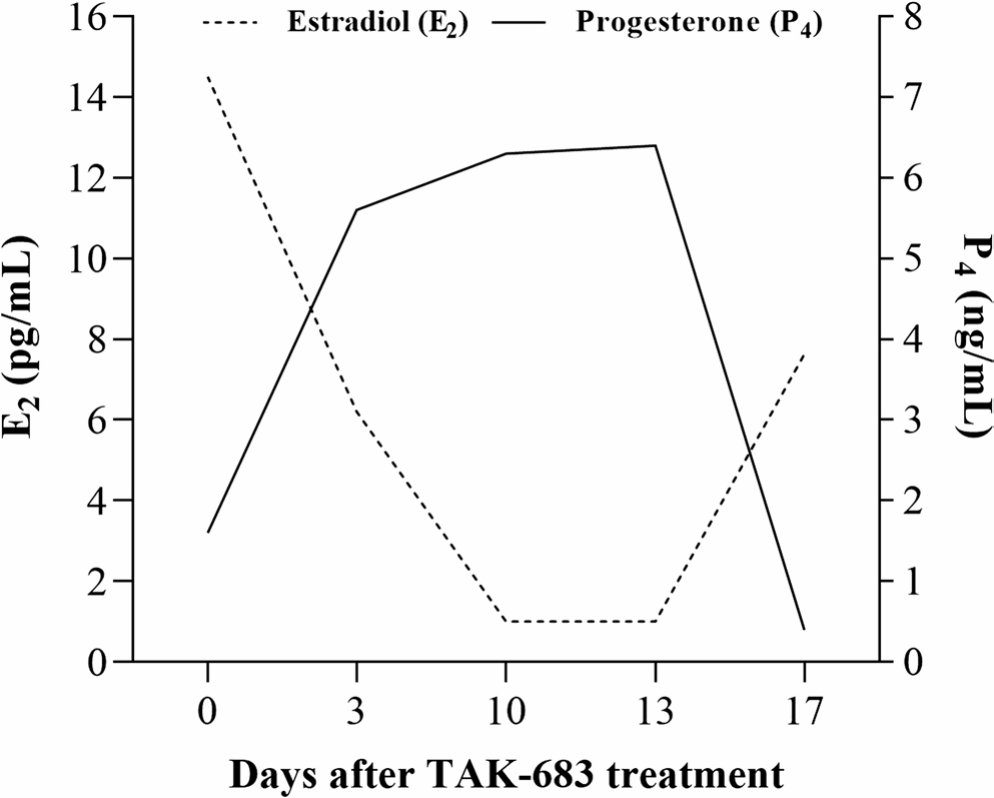
Temporal changes in serum estradiol (E_2_, pg/mL) and progesterone (P_4_, ng/mL) concentrations after a single subcutaneous administration of 5 µg of TAK-683 for the treatment of a cystic follicle diagnosed in an Aleppo lactating goat following a synchronized estrus induction protocol during the non-breeding season

The blood samples were centrifuged at 3000 rpm for 10 min to obtain serum. Serum E_2_ and P_4_ concentrations were measured using a chemiluminescence immunoassay analyser (CLIA) (MAGLUMI-X8, Snibe Diagnostic, China). The intra- and inter-assay coefficients of variation (CV) were 1.50–6.84% and 1.87–5.05% for E_2_, and 2.02–4.33% and 1.83–7.21% for P_4_, respectively. Hormonal analysis revealed elevated E_2_ (14.5 pg/mL) and low P_4_ (1.6 ng/mL) levels, confirming the diagnosis of a follicular cyst (Fig. 3).

### Treatment

For therapeutic purposes, a long-acting kisspeptin analog, TAK-683 (purity ≥ 99.91%, TAK-683 acetate, MedChemExpress^®^, USA, Cat. No: HY-P2161B), was used. The lyophilized peptide was reconstituted in sterile phosphate-buffered saline (pH 7.4, PBS solution, Testonic Laboratory, Colin Chemical Industry, Türkiye, Cat. No: 8680131853229) to prepare an injectable solution at a final concentration of 5 µg/mL (equivalent to 3.680 µM). The solution was prepared under aseptic conditions and administered on the same day. The treatment dose was selected based on previously established protocols in goats (Kanai et al. 2017) and ewes (Uyanık et al. 2024). Accordingly, a single subcutaneous injection of 1 mL containing 5 µg of TAK-683 was administered (Fig. 1).

### Post-treatment hormonal profile and ovarian changes

Blood samples were taken from the jugular vein on days 3, 10, 13, and 17 after treatment to monitor changes in serum E_2_ and P_4_ levels. The sera were separated and stored at -20 °C until analysis. In addition, on the same days as blood sampling, both B-mode and color Doppler transrectal ultrasonography were performed to evaluate the ovaries (Fig. 1). In B-mode scans, follicular and luteal structures were measured, and their diameters (mm) and surface areas (mm²) were calculated. In color Doppler mode, the vascularized area of the luteal tissue was determined, and a vascularization index (VI) was calculated using the following formula: VI (%) = Area with Doppler signal (mm²) / Total luteal area (mm²) × 100.

On Day 3 post-treatment, serum E_2_ level decreased to 7.2 pg/mL, while P_4_ increased to 5.6 ng/mL (Fig. 3). Ultrasonography revealed that the follicular diameter had decreased to 15.4 mm, whereas wall thickness increased to 2.58 mm (Fig. 2B). Behavioural observations indicated a complete disappearance of nymphomaniac signs on this day. By Day 10, partial luteinization of the cystic follicle was observed. Serum E_2_ dropped to 1.5 pg/mL, and P_4_ rose to 6.3 ng/mL. At this stage, the CL had a diameter of 14.18 mm, a surface area of 94.86 mm², and a VI of 21.54% (Fig. 2C). On Day 13, the CL reached its maximum development with a diameter of 15.04 mm, a surface area of 129.24 mm², and a VI of 28.12% (Fig. 2D). Parallel to the ultrasonographic findings, the highest serum P_4_ level was recorded at 6.4 ng/mL, while E_2_ remained at basal levels (Fig. 3). Finally, by Day 17, spontaneous luteolysis had occurred: P_4_ dropped to 0.4 ng/mL, E_2_ increased to 7.63 pg/mL, and three small follicles (< 5 mm) were detected in the contralateral ovary (Fig. 2E). Finally, serial ultrasonographic examinations showed no CL formation in the right ovary, and the small follicles on that side exhibited no evidence of ovulation or luteinization. In contrast, the follicle accompanying the cystic follicle in the left ovary underwent luteinization.

## Discussion

Follicular cysts in goats represent a significant gynaecological disorder negatively affecting reproductive performance, with a prevalence of approximately 15–20% (Medan et al. 2004; Khan et al. 2016; Samuel et al. 2025). The underlying cause of a follicular cyst is the failure of ovulation, which is most often associated with an inadequate LH surge required to trigger ovulation (Ortega et al. 2015). In addition, out-of-seasonal breeding, poor nutrition, and lactation are physiological stressors that may further increase the risk of ovulatory failure (Silva et al. 2020). Moreover, it has been reported that due to its long half-life, eCG used in synchronization protocols may sustain the development of multiple follicles under anestrous conditions; however, if the preovulatory LH surge is delayed, some of these follicles may fail to ovulate and persist (Hervé et al. 2004). The 500 IU eCG dose used in this case is close to the upper limit of the commonly reported ranges for goats; however, it is widely used as a standard dose during anestrus (Véliz-Deras et al. 2020; Habibizad et al. 2020; Sharif et al. 2023). Furthermore, in our literature review, we did not identify any reports indicating that eCG administered at similar (Regueiro et al. 1999) or even higher doses (Mohtar et al. 2014) induces ovarian cyst formation in goats. On the other hand, no comparable cystic development was observed in other goats exposed to the same synchronization protocol and eCG dose. Therefore, it is thought that the follicular cyst observed in this case is more related to multifactorial physiological factors such as the goat being out of breeding season, being in lactation, and LH deficiency resulting from possible metabolic stress conditions, rather than the eCG dose.

The diagnosis of follicular cysts is based on a combined assessment of ultrasonographic imaging and hormonal profile findings. In our case, transrectal ultrasonography revealed an anechoic follicular cyst measuring > 10 mm in diameter in the ovary, and the absence of an active CL further supported the diagnosis. Typically, follicular cyst cases are characterized by elevated E_2_ concentrations and low P_4_ levels due to the lack of luteal tissue (Simões et al. 2009). Consistent with the literature, this case also exhibited high E_2_ levels associated with nymphomaniac estrus behaviour and basal P_4_ concentrations. The increased serum E_2_ levels explained the repeated estrus signs (nymphomania) observed in the goat (Samuel et al. 2025). Taken together, these findings indicate that the diagnosis of follicular cysts can be reliably established through the combined interpretation of clinical signs (prolonged estrus), ultrasonographic evidence, and hormonal evaluation. Indeed, transrectal ultrasonography has been reported to be effective in detecting ovarian cystic structures in goats, and when combined with E_2_ measurements, diagnostic accuracy is further improved (Medan et al. 2004; Simões et al. 2009).

The use of TAK-683 in treatment represents an innovative approach in terms of its mechanism of action. Kisspeptin and its receptor (KISS1R) are key regulators of reproduction in mammals, triggering GnRH release from the hypothalamus and thereby initiating reproductive functions (Beltramo and Decourt 2018). TAK-683 is a synthetic decapeptide analog developed to mimic the C-terminal region of kisspeptin, which activates GnRH neurons and induces the secretion of LH and FSH from the pituitary (Kanai et al. 2017; Uyanık et al. 2024). Experimental evidence indicates that TAK-683 may exert differential effects on gonadotropin secretion depending on the mode of administration. Tanaka et al. (2013) demonstrated that chronic exposure to TAK-683 suppresses pulsatile LH secretion in ovariectomized goats while preserving the capacity to generate an E_2_-induced preovulatory LH surge. In contrast, acute administration of TAK-683 has been shown to stimulate surge-type LH release (Kanai et al. 2017). This pharmacological effect ultimately stimulates gonadotropin release, promoting follicular maturation, ovulation, and/or luteinization (Goto et al. 2014; Uyanık et al. 2024). In our case, administration of TAK-683 successfully triggered ovulation/luteinization of the follicular cyst, as expected. Consistently, previous studies have reported that a single dose 5 µg of TAK-683 administered during the follicular phase in goats induced an LH surge within approximately 4 h, followed by ovulation within 3 days (Kanai et al. 2017).

The clinical outcome of the present case demonstrated that TAK-683 provided an effective therapeutic response. Following treatment, nymphomaniac estrus signs disappeared, and progesterone levels rose to luteal phase values within a few days, indicating luteinization of the cyst. GnRH agonists have previously been shown to be effective in the treatment of follicular cysts by inducing a strong LH surge and subsequent ovulation ( Medan et al. 2004; Khan et al. 2016). Similarly, the endocrine responses and ultrasonographic findings observed after TAK-683 administration paralleled those reported with GnRH treatments, suggesting that TAK-683 could represent a potential alternative to conventional GnRH therapy in the management of follicular cysts. Overall, this case highlights that TAK-683 may not only be useful for ovulation synchronization in small ruminants but could also offer a promising therapeutic option for gynaecological conditions such as follicular cysts. Nevertheless, conclusions cannot be generalized from a single case, and further controlled studies are required to determine the optimal dosage, treatment protocols, long-term reproductive outcomes, and comparative efficacy against GnRH analogs.

## Data Availability

The complete data associated with this manuscript are provided within the article.
